# Supplementary material for: Structural basis for Gemin5 decamer-mediated mRNA binding
Source: Nat Commun. 2022 Sep 2;13:5166. doi: 10.1038/s41467-022-32883-z (PMC9440017; doi:10.1038/s41467-022-32883-z)
Supplement: Supplementary file 1 — Supplmentary Information [file 41467_2022_32883_MOESM1_ESM.pdf]

Supplementary Information

Structural basis for Gemin5 decamer-mediated mRNA binding

Qiong Guo[1,#], Shidong Zhao[1,#], Rosario Francisco-Velilla[2,#], Jiahai Zhang[1], Azman Embarc-Buh[2], Salvador Abellan[2], Mengqi Lv[1], Peiping Tang[1], Qingguo Gong[1], Huaizong Shen[3], Linfeng Sun[1], Xuebiao Yao[1], Jinrong Min[4,5], Yunyu Shi[1], Encarnacion Martínez-Salas[2,*], Kaiming Zhang[1,*], Chao Xu[1,*]

[1]MOE Key Laboratory for Cellular Dynamics, School of Life Sciences, Division of Life Sciences and Medicine, University of Science and Technology of China, 230027, Hefei, P.R. China;

[2]Centro de Biología Molecular Severo Ochoa, CSIC-UAM, Nicolás Cabrera 1, 28049 Madrid, Spain;

[3]Key Laboratory of Structural Biology of Zhejiang Province, School of Life Sciences, Westlake University, Hangzhou, Zhejiang 310024, China

[4]Structural Genomics Consortium, University of Toronto, Toronto, Ontario M5G 1L7, Canada;

[5]Department of Physiology, University of Toronto, Toronto, Ontario M5S 1A8, Canada.
#These authors contributed equally

*To whom correspondence should be addressed. E-mail: emartinez@cbm.csic.es; kmzhang@ustc.edu.cn; xuchaor@ustc.edu.cn

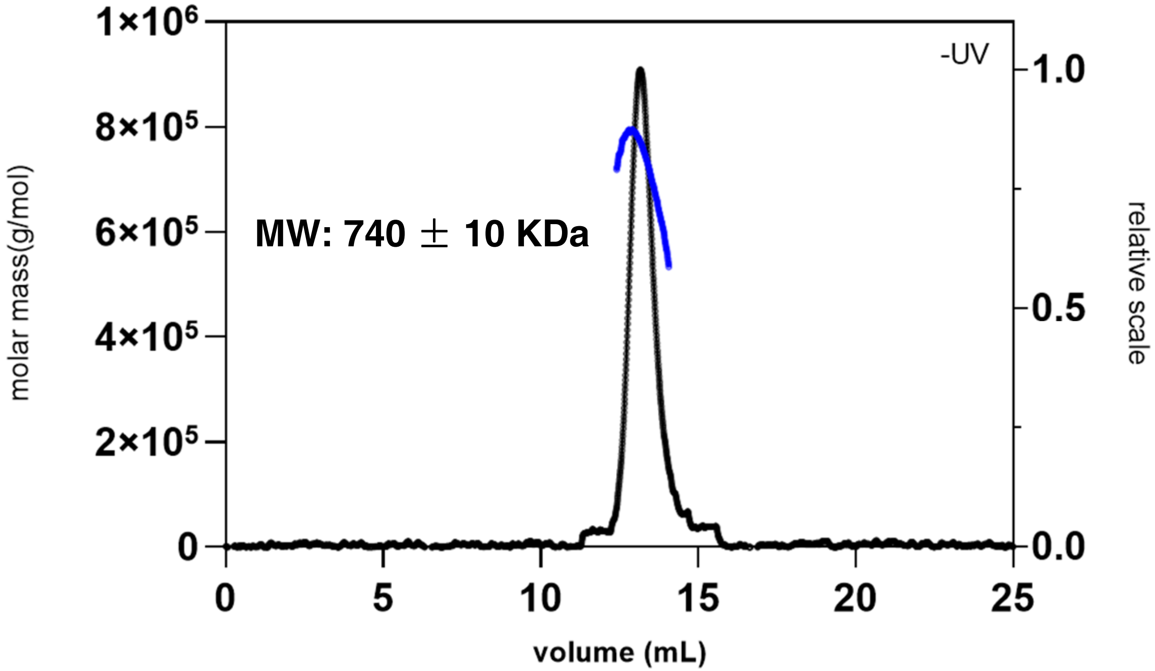

Supplementary Figure 1 Static light scattering (SLS) experiment indicates the molecular weight of recombinant G5C is 740 $\pm$ 10 KDa in solution.

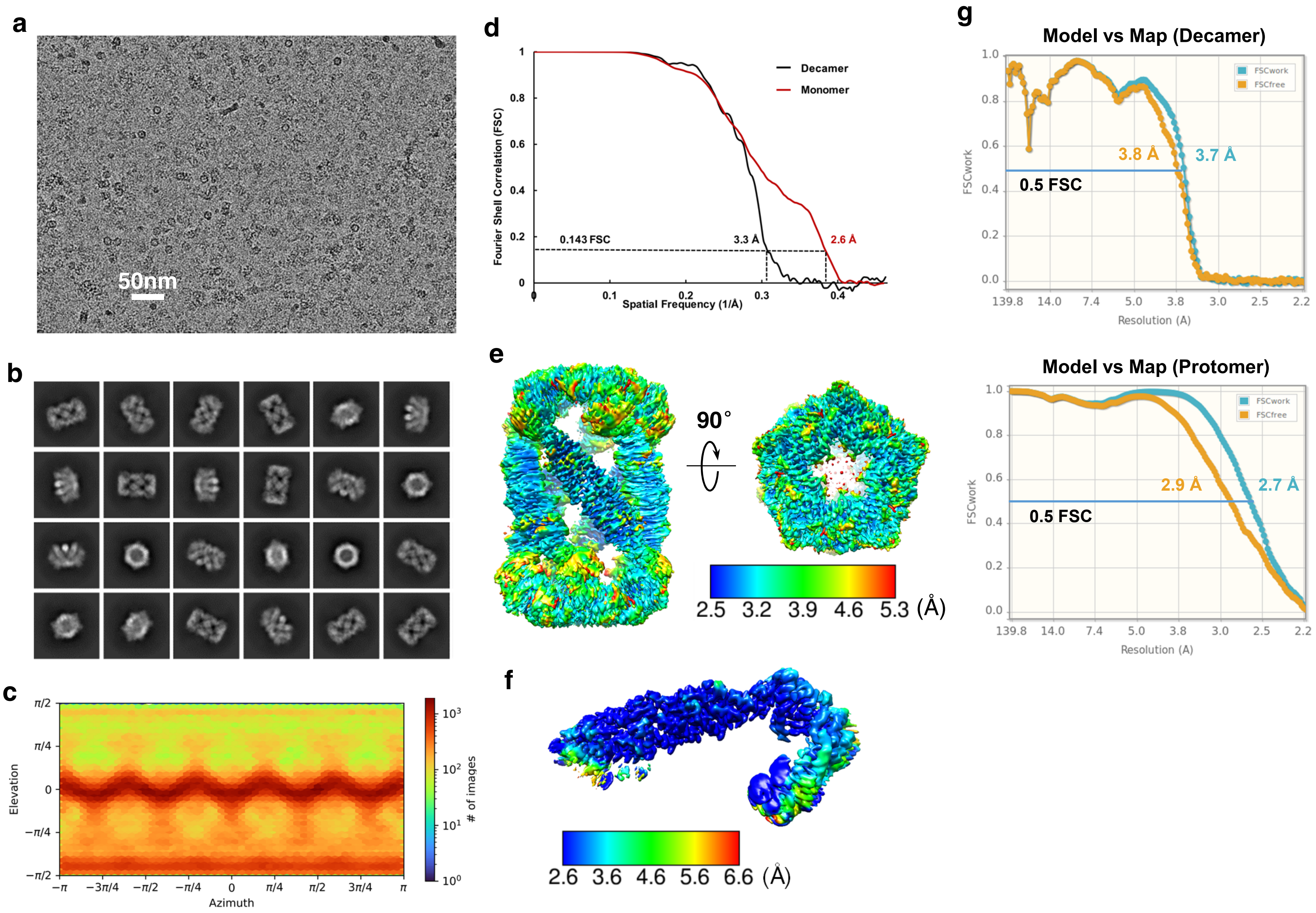

a
50nm
b
c
π/2
π/4
0
−π/4
−π/2
Elevation
−π
−3π/4
−π/2
−π/4
0
π/4
π/2
3π/4
π
Azimuth
10^3
10^2
10^1
10^0
# of images
d
Fourier Shell Correlation (FSC)
Spatial Frequency (1/Å)
Decamer
Monomer
0.143 FSC
3.3 Å
2.6 Å
e
90°
2.5 3.2 3.9 4.6 5.3 (Å)
f
2.6 3.6 4.6 5.6 6.6 (Å)
g
Model vs Map (Decamer)
FSCwork
FSCfree
3.8 Å
3.7 Å
0.5 FSC
Resolution (A)
139.8 14.0 7.4 5.0 3.8 3.0 2.5 2.2
Model vs Map (Protomer)
2.9 Å
2.7 Å
0.5 FSC

Supplementary Figure 2 Cryo-EM analysis of the human G5C. **a** Representative cryo-EM image of G5C after motion correction and contrast transfer function (CTF) correction. The images were processed independently at least twice with similar results. **b** Representative 2D class averages of G5C. **c** Distribution of particle angles calculated in cryoSPARC for projections. **d** The gold-standard Fourier shell correlation curve for the overall map. Black refers to the overall structure of the decamer. Red refers to the monomer structure obtained after symmetry expansion and local refinement. **e-f** The density maps colored by local resolution estimation, which is calculated by CryoSPARC, using UCSF Chimera. **g** The FSC plots of half maps compared with the refined model (only half map 1 was used for refinement) . FSCwork, model vs half map 1; FSCfree, model vs half map 2.

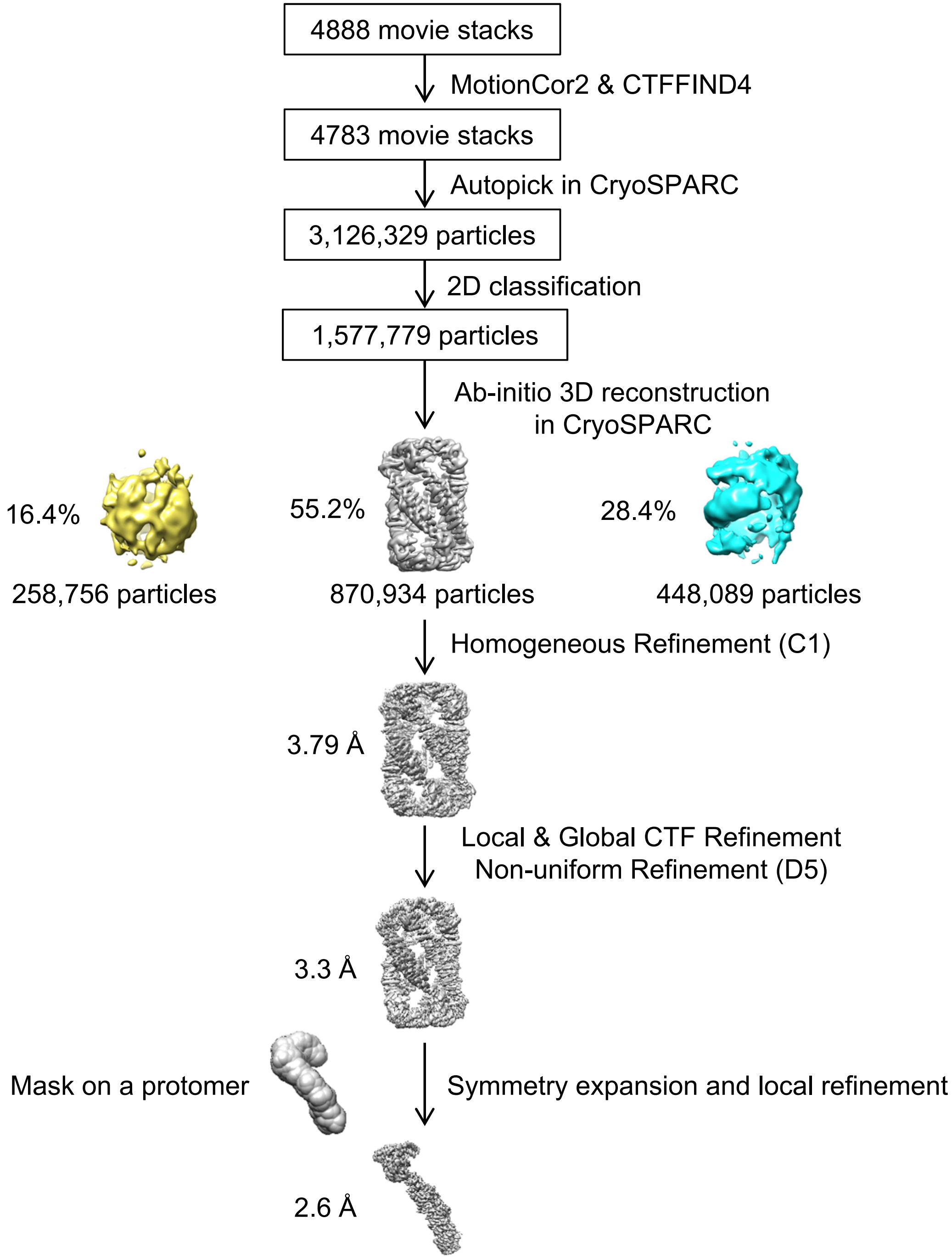

Supplementary Figure 3 Flowchart for cryo-EM data processing of human G5C.

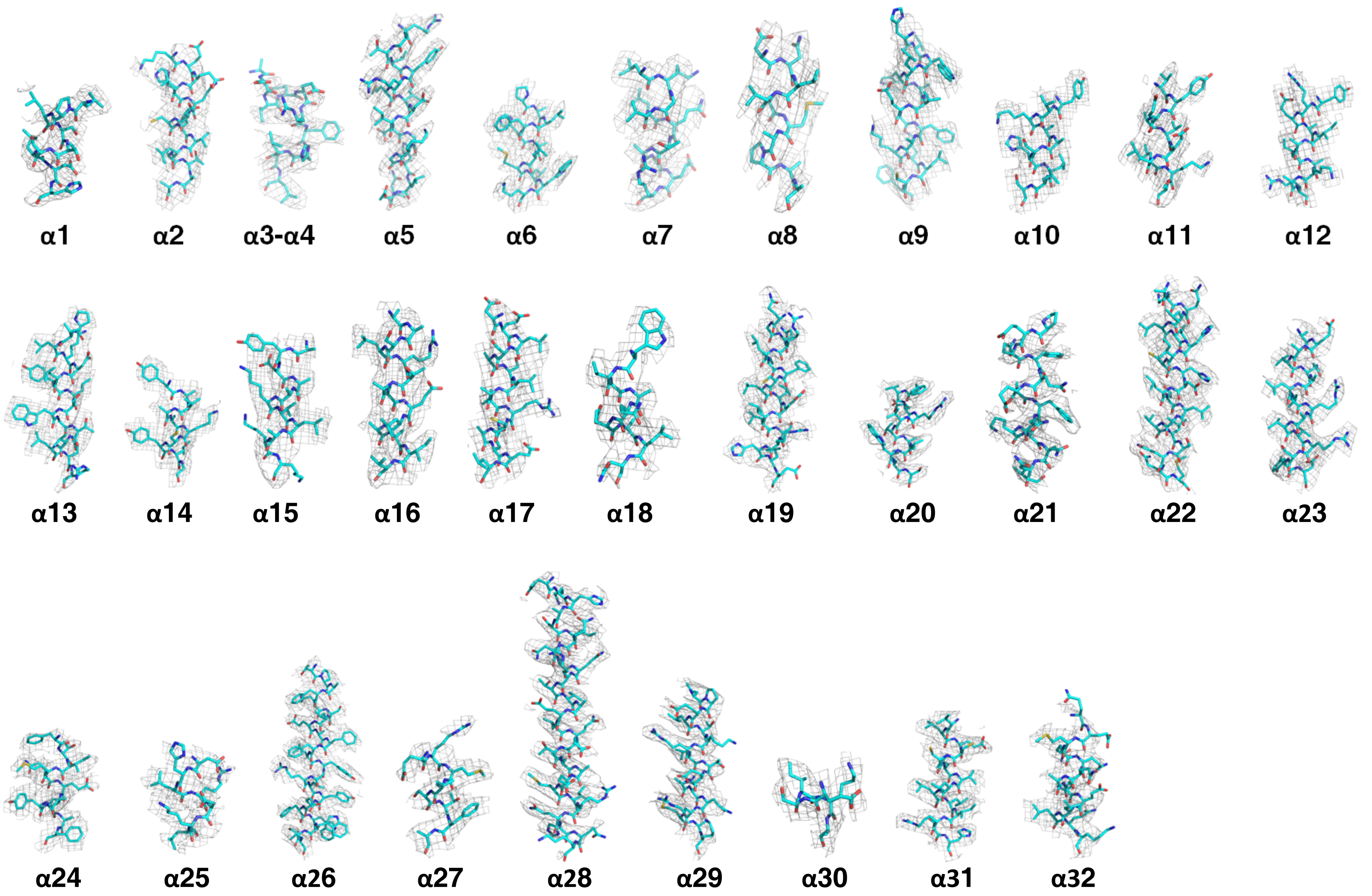

Supplementary Figure 4 Cryo-EM density maps of representative segments of human G5C, including α1-α32, are contoured at 5.0 σ.

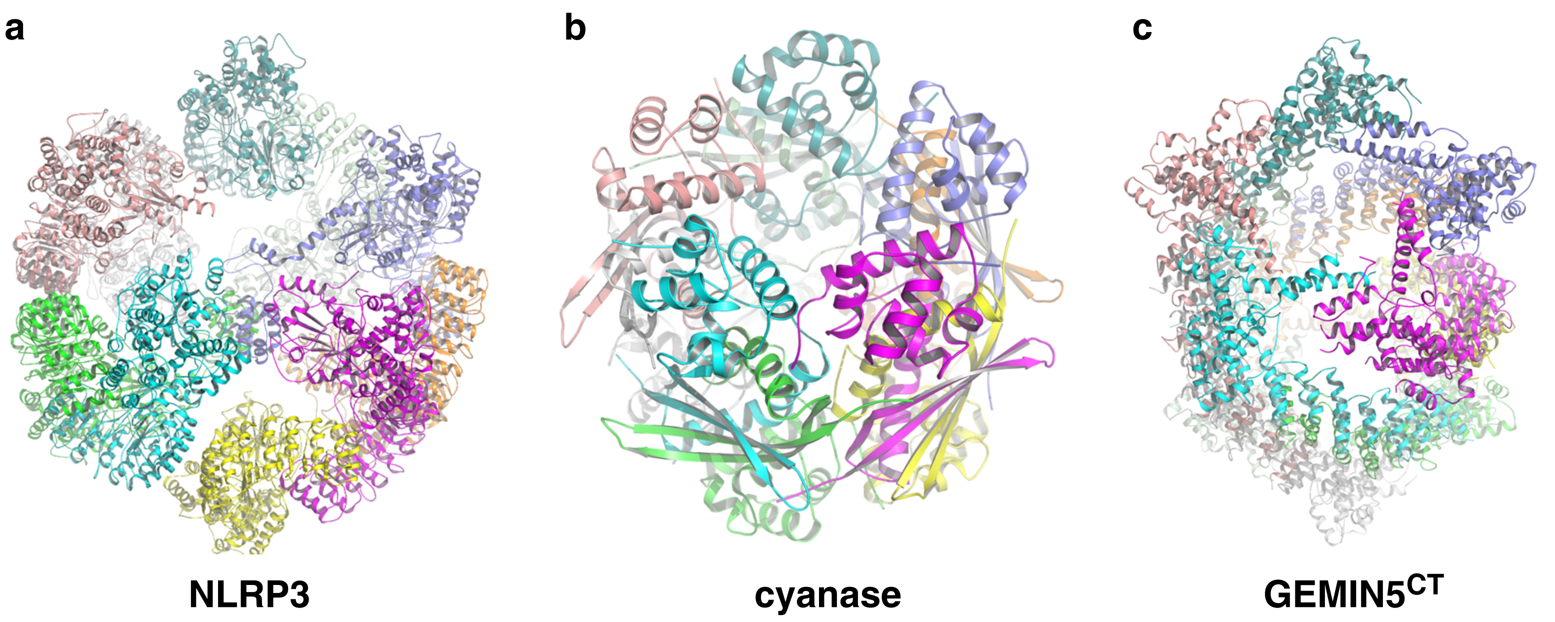

Supplementary Figure 5 Structure comparison of three homodecamers, including **a** NLRP3 (PDB id: 7PZC), **b** cyanase (PDB id: 1DW9), and **c** Gemin5^{CT}. The color mode is the same for three decamers.

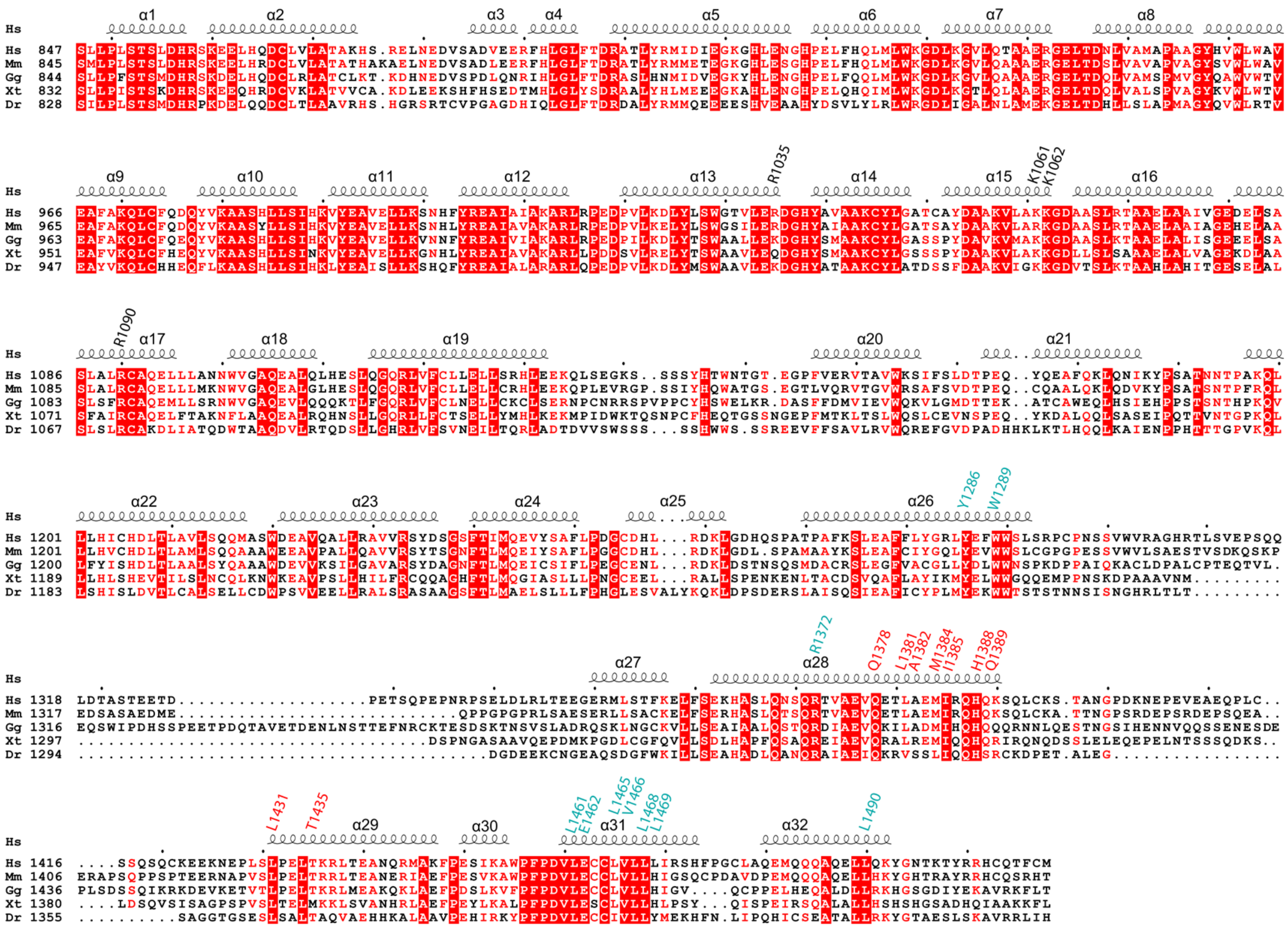

Supplementary Figure 6 Sequence alignment of human G5C and its orthologs, including G5C from *Homo sapiens* (Hs, NP_001239085.1), *Mus musculus* (Mm, NP_001160141.1), *Gallus gallus* (Gg, XP_015149397.1), *Xenopus tropicalis* (Xt, XP_004912843.1), and *Danio rerio* (Dr, XP_001339880.4). The secondary structures of human G5C are labelled at the top of the sequences. Residues at the pentamer interface are labeled in the same way as shown in Fig. 3d, with residues in molecule A and B, colored in red and cyan, respectively. The four basic residues within TPR that are spatially proximal to RBS1, are labelled in black.

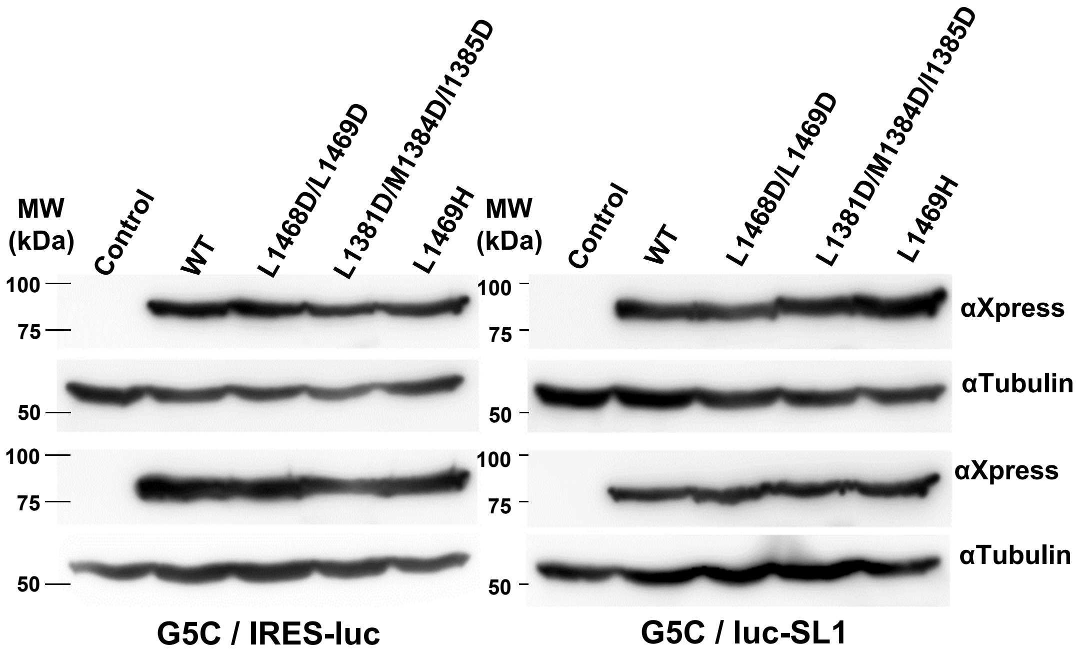

Supplementary Figure 7. Western blot analysis of biological replicates used in translation assays. Protein expression in HEK293 cells was monitored by WB using anti-Xpress for G5C proteins, tubulin was used as loading control.

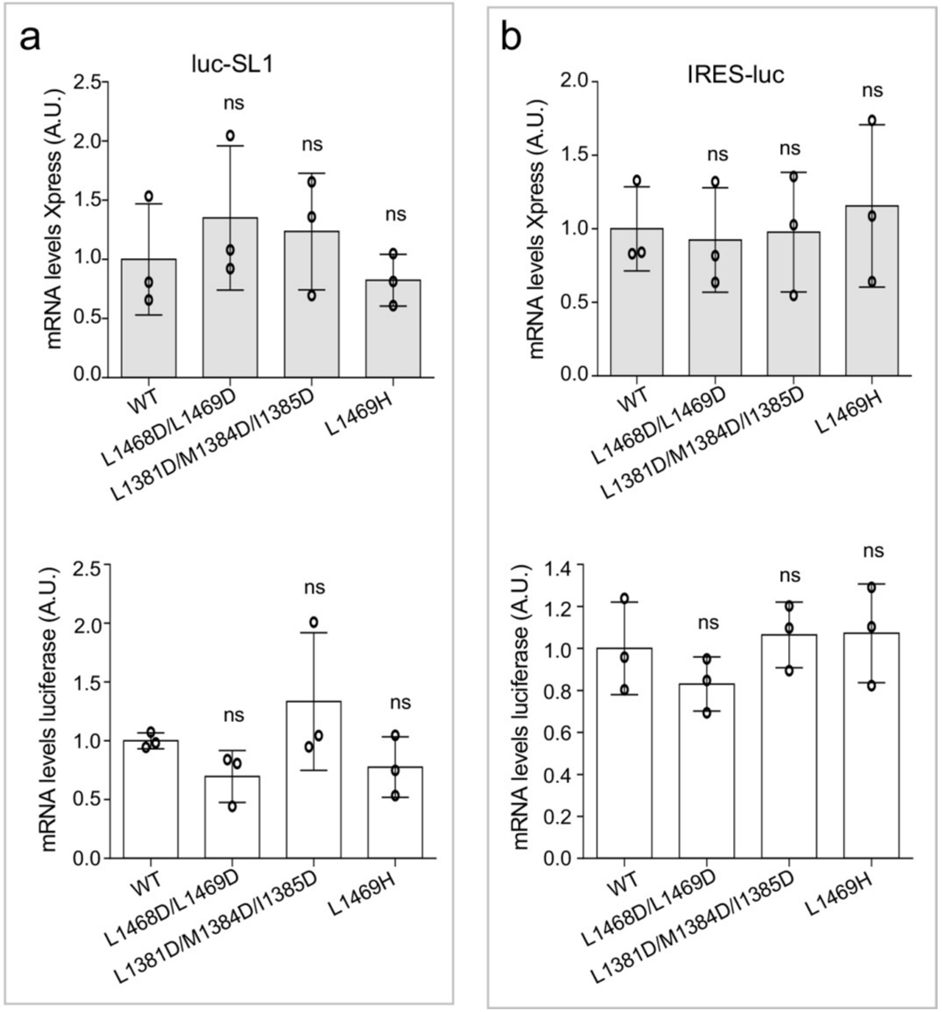

Supplementary Figure 8. Steady-state mRNA levels of Xpress-G5C (grey bars) and luciferase reporter (white bars) present in transfected cells at the time of harvesting determined by RTqPCR for **a** (luc-SL1) and **b** (IRES-luc) translation. Values represent the mean ± SD. ns, not significant; n = 3 replicates; two-tailed paired t-tests. The P values are 0.474395, 0.579970, 0.587735 for Xpress mRNA levels and 0.085561, 0.382091, 0.219987 for luciferase reporter mRNA levels in **a** (luc-SL1). P values are 0.788559, 0.940065, 0.687438 for Xpress mRNA levels and 0.314304, 0.701428, 0.717109 for luciferase reporter mRNA levels in **b** (IRES-luc).

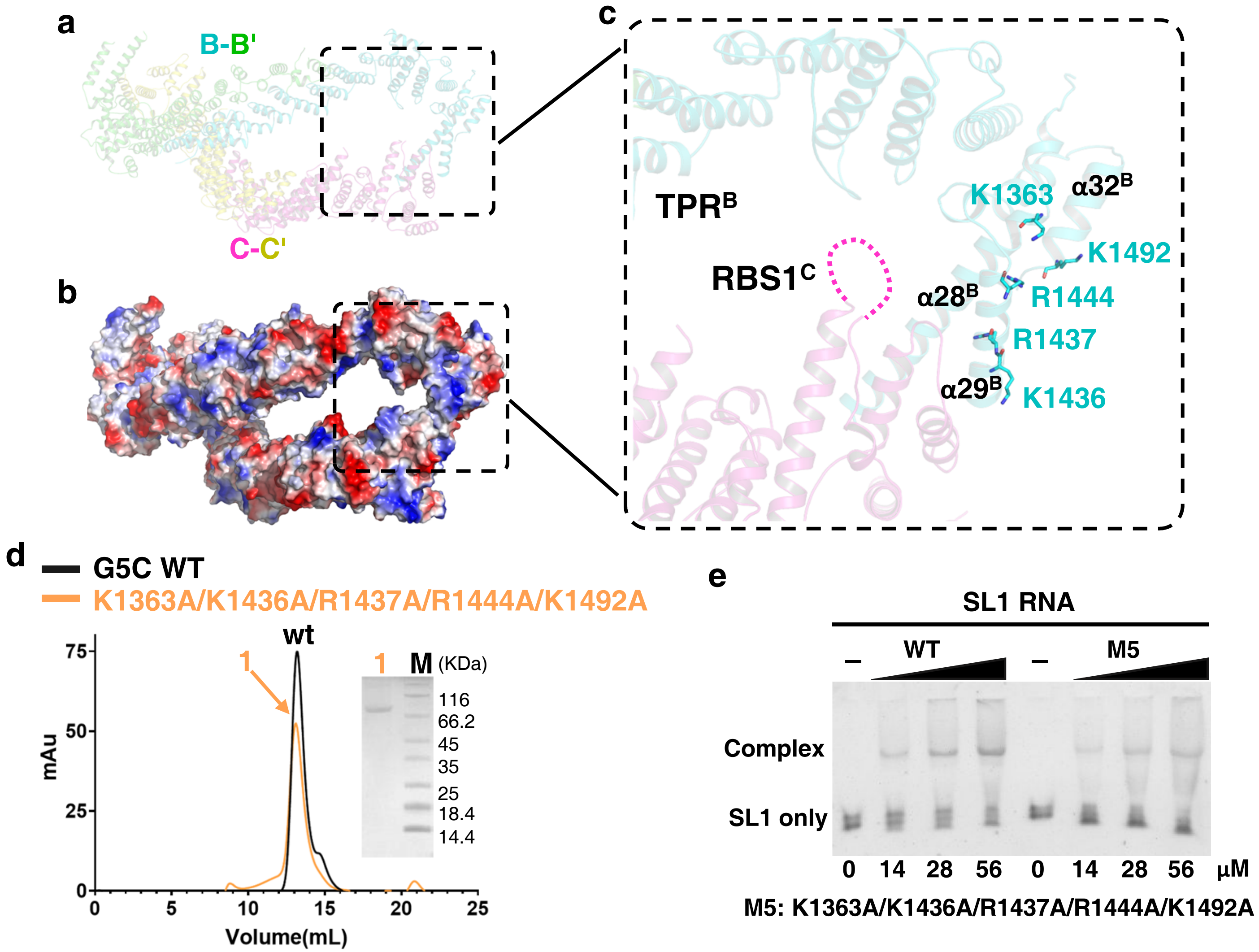

Supplementary Figure 9 Relevance of the quintuple mutation site with RNA binding concave. a B-B' and C-C' are two spatially adjacent dimers. G5C molecules are shown as in Fig. 6a. **b** Electrostatic surface of the two adjacent dimers. **c** Close-up view of the quintuple mutation site. The five basic residues in α28^{B}, α29^{B} and α32^{B}, including K1363, K1436, R1437, R1444, and K1492, are shown in sticks. The TPR^{B} domain and RBS1^{C} region are indicated. The invisible loops within RBS1^{C} is indicated by dashes. **d** SEC and **e** EMSA binding assay for quintuple mutant K1363A/K1436A/R1437A/R1444A/K1492A. For Supplementary Fig. 9d-9e, each experiment was repeated independently twice with similar results.

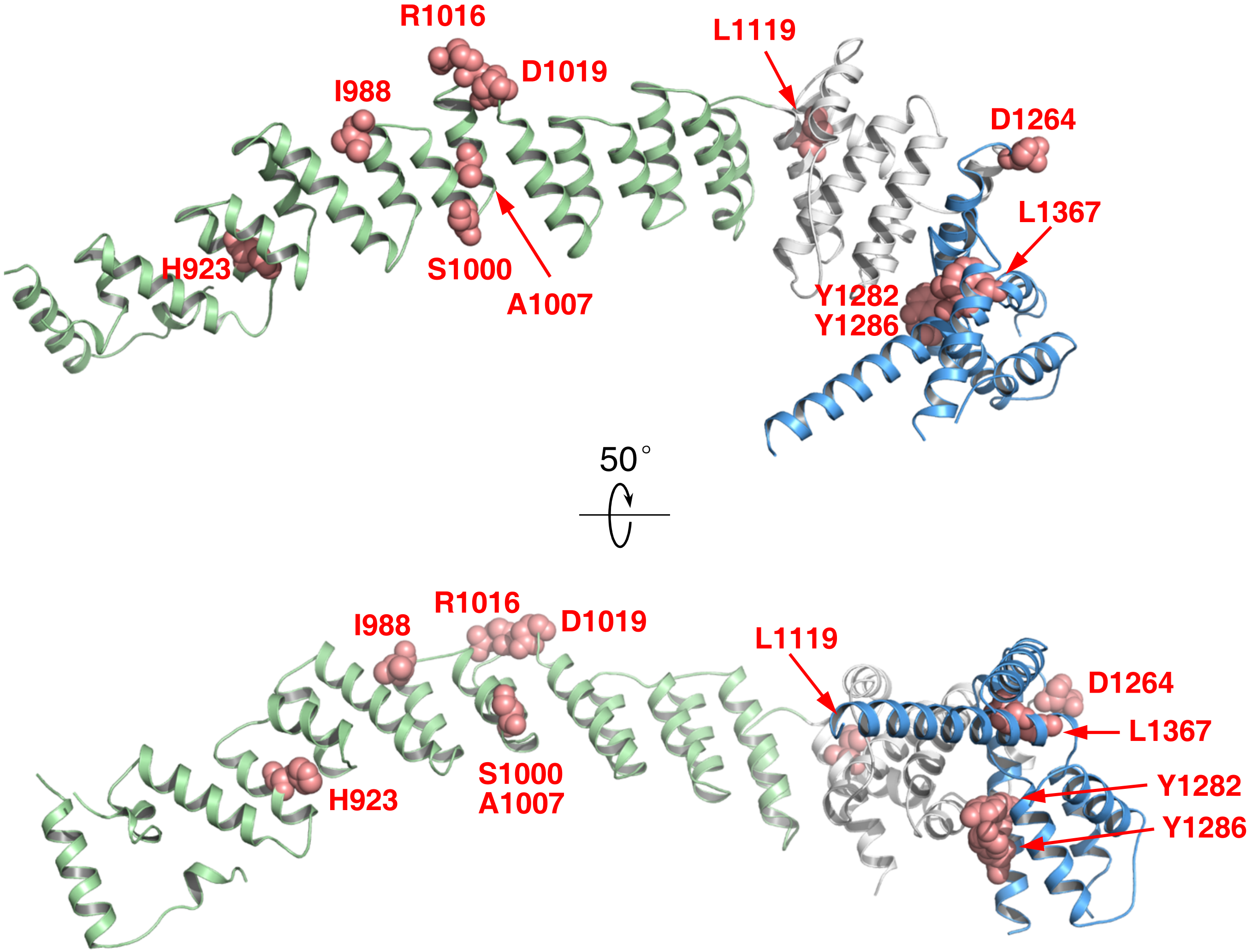

Supplementary Figure 10 G5C mutations associated with neurodevelopmental disorder. The structure of G5C is colored in the same way as shown in Fig. 2a. Reported G5C mutations associated with neurodevelopmental disorder, are labeled and shown in red spheres

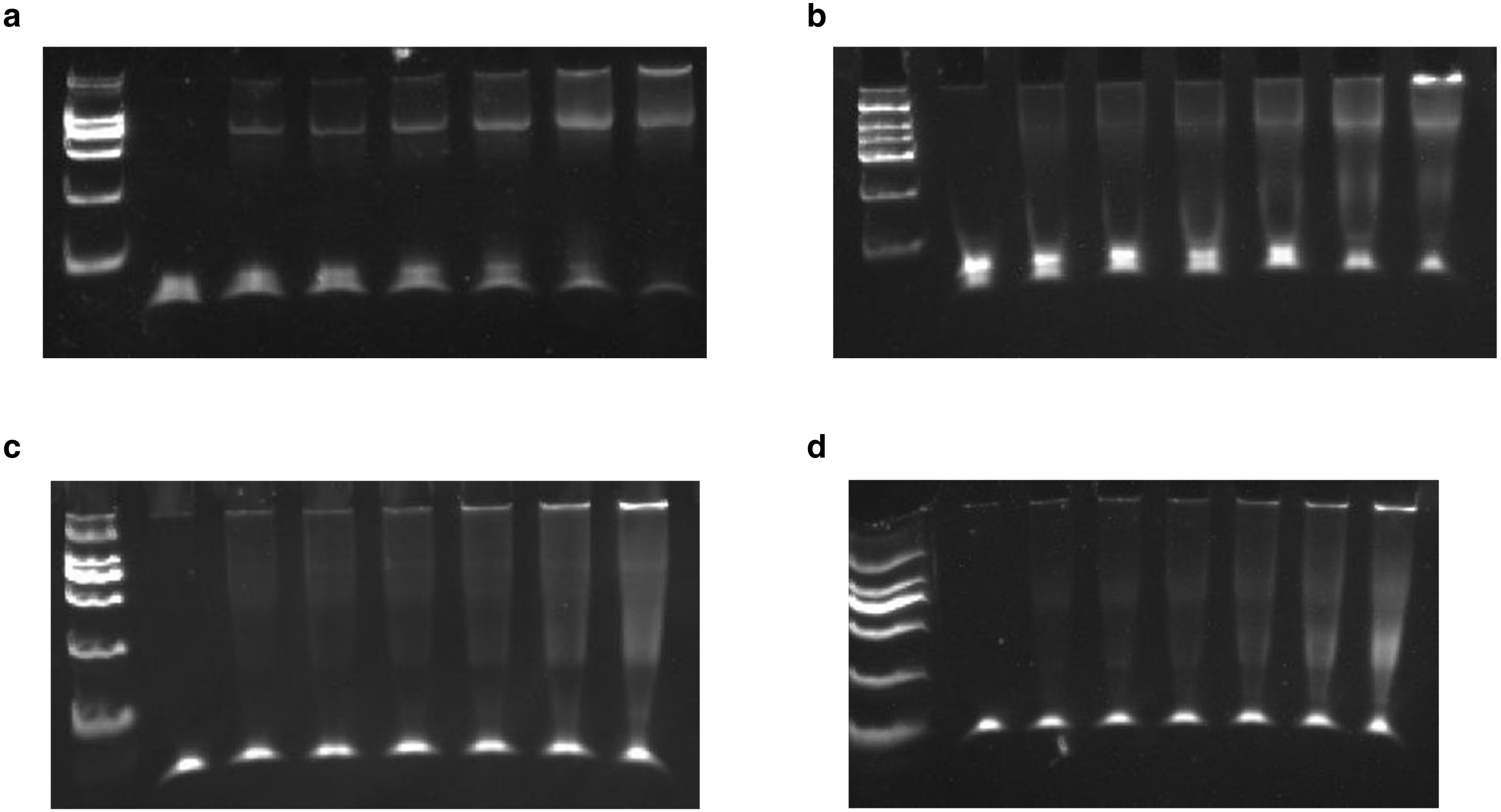

Supplementary Figure 11 Original Gel figures for duplicate EMSA. **a** #1 for G5C binding to SL1; **b** #2 for G5C binding to SL1; **c** #1 for G5C binding to D5-IRES; **d** #2 for G5C binding to D5-IRES.

Supplementary Table 1. Cryo-EM data collection, refinement and validation statistics

	Gemin5$_{841-1508}$ decamer	Gemin5$_{841-1508}$ protomer
Magnification	81000	81000
Voltage (kV)	300	300
Exposure rate (e^-/ Å^2)	8	8
Defocus range (μm)	-2.5 - -1.5	-2.5 - -1.5
Pixel size (Å)	1.07	1.07
Super-resolution mode	Yes	Yes
Symmetry imposed	D5	C1
Initial particle images (no.)	3126329	3126329
Final particle images (no.)	870934	8709340
Map resolution (Å)	3.31	2.60
FSC threshold	0.143	0.125
Map resolution range (Å)	2.50 - 5.30	2.60 - 6.60
Initial model used (PDB code)	6RNQ	6RNQ
Model resolution (Å)	3.31	2.60
FSC threshold	0.143	0.143
**Model composition**		
Nonhydrogen atoms	41990	4340
Protein residues	5310	546
**Validation**		
R.m.s. deviations		
Bond lengths (Å)	0.004	0.004
Bond angles (°)	0.98	0.73
MolProbity score	1.93	2.13
Clashscore	11.26	8.47
Poor rotamers (%)	0.5	0.4
Ramachandran plot		
Favored (%)	94.8	93.7
Allowed (%)	5.2	6.3
Disallowed (%)	0	0

Supplementary Table 2. Primers used for binding experiments

Primers	Sequences (5' to 3')
R904A/K911A/K933A/R942A_1	GGCTACCCTGTATGCAATGATTGATATTGAAGGAGCAGGTCACTTAGAAAATG
R904A/K911A/K933A/R942A_2	TTTCTAAGTGACCTGCTCCTTCAATATCAATCATTGCATACAGGGTAGCCCTG
R904A/K911A/K933A/R942A_3	GCTTTGGAAAGGAGATCTCGCAGGTGTTCTCCAGACTGCAGCAGAAGCAGGGGAGCTG
R904A/K911A/K933A/R942A_4	TCTGTCAGCTCCCCTGCTTCTGCTGCAGTCTGGAGAACACCTGCGAGATCTCCTTTCC
R1035A/K1061A/K1062A/R1090A_1	GCTGGGGAACCGTCCTAGAAGCAGATGGCCACTATGCTGTAG
R1035A/K1061A/K1062A/R1090A_2	GGCAGCTACAGCATAGTGGCCATCTGCTTCTAGGACGGTTCC
R1035A/K1061A/K1062A/R1090A_3	GATGCAGCCAAAGTTTTGGCCGCAGCAGGGGATGCGGCATCACTTAG
R1035A/K1061A/K1062A/R1090A_4	CGTTCTAAGTGATGCCGCATCCCCTGCTGCGGCCAAAACTTTGGCTG
R1035A/K1061A/K1062A/R1090A_5	GTTGTCTGCTTCCCTGGCTCTCGCATGTGCCCAAGAGCTGCTTCTG
R1035A/K1061A/K1062A/R1090A_6	GGCCAGAAGCAGCTCTTGGGCACATGCGAGAGCCAGGGAAGCAGAC
K1363A/K1436A/R1437A/R1444A/K1492A_1	GTACTTTTAAGGAGCTCTTTTCAGAAGCACATGCCAGTCTCCAAAACTCAC
K1363A/K1436A/R1437A/R1444A/K1492A_2	TCTGTGAGTTTTGGAGACTGGCATGTGCTTCTGAAAAGAGCTCCTTAAAAG
K1363A/K1436A/R1437A/R1444A/K1492A_3	CTCTGCCTGAGTTAACCGCAGCACTTACCGAGGCAAATCAGGCAATGGCAAAATTTC
K1363A/K1436A/R1437A/R1444A/K1492A_4	CAGGAAATTTTGCCATTGCCTGATTTGCCTCGGTAAGTGCTGCGGTTAACTCAGGC
K1363A/K1436A/R1437A/R1444A/K1492A_5	GCAGGCCCAAGAGCTCCTTCAGGCATACGGCAACACGAAAACTTAC
K1363A/K1436A/R1437A/R1444A/K1492A_6	GTCTTCTGTAAGTTTTCGTGTTGCCGTATGCCTGAAGGAGCTCTTG
L1468D/L1469D_1	GTGCTGGAGTGCTGCCTCGTCCTGGATGATATCAGGTCCCACTTTCCTGGCTGTC
L1468D/L1469D_2	CCAGGAAAGTGGGACCTGATATCATCCAGGACGAGGCAGCACTCCAGCACATCTG
L1381D/M1384D/I1385D_1	GTTGCTGAAGTCCAAGAGACCGATGCAGAAGATGATCGACAACACCAAAAGAGTCAAC
L1381D/M1384D/I1385D_2	GAGTTGACTCTTTTGGTGTTGTCGATCATCTTCTGCATCGGTCTCTTGGACTTCAGC
L1469H_1	GCTGGAGTGCTGCCTCGTCCTGCTTCATATCAGGTCCCACTTTCCTGGCTGTC
L1469H_2	CCAGGAAAGTGGGACCTGATATGAAGCAGGACGAGGCAGCACTCCAGCACATC
A951E_1	GAGGGGAGCTGACAGACAACCTTGTGGAAATGGCACCAGCAGCTGGCTACCATG
A951E_2	GTAGCCAGCTGCTGGTGCCATTTCCACAAGGTTGTCTGTCAGCTCCCCTCTTTC

Supplementary Table 3. **Primers used for translation assays**

Primers	Sequences (from 5' to 3')
L1381D_s	GTGTTGTCGGATCATTTCTGCGTCGGTCTCTTGGACTTCAGCAAC
L1381D_as	GTTGCTGAAGTCCAAGAGACCGACGCAGAAATGATCCGACAACAC
M1384D/I1385D_s	GTTGACTCTTTTGGTGTTGTCGGTCGTCTTCTGCGTCGGTCTCTTGGACTTCAG
M1384D/I1385D_as	CTGAAGTCCAAGAGACCGACGCAGAAGACGACCGACAACACCAAAAGAGTCAAC
L146H_s	AGTGGGACCTGATGTGAAGCAGGACGAGG
L146H_as	CCTCGTCCTGCTTCACATCAGGTCCCACT
L1468DL1469D_s	GGAAAGTGGGACCTGATGTCATCCAGGACGAGGCAGCACTC
L1468DL1469D_as	GAGTGCTGCCTCGTCCTGGATGACATCAGGTCCCACTTTCC

Supplementary Table 4. Quantitation from EMSA assay for G5C binding to SL1 and IRES RNAs.

G5C Concentrations (μM)	Fraction bound SL1 (#1)	Fraction bound SL1 (#2)	Fraction bound D5-IRES (#1)	Fraction bound D5-IRES (#2)
56	0.876	0.812	0.581	0.470
28	0.693	0.620	0.341	0.287
14	0.534	0.383	0.182	0.127
7	0.3760	0.308	0.085	0.063
3.5	0.247	0.246	0.056	0.072
1.75	0.258	0.183	0.048	0.044
0	0	0	0	0
