# Supplementary material for: Structural basis for Gemin5 decamer-mediated mRNA binding
Source: Nat Commun. 2022 Sep 2;13:5166. doi: 10.1038/s41467-022-32883-z (PMC9440017; doi:10.1038/s41467-022-32883-z)
Supplement: Supplementary file 3 — Reporting Summary [file 41467_2022_32883_MOESM3_ESM.pdf]

nature portfolio

Corresponding author(s): Chao Xu

Last updated by author(s): Aug 15, 2022

Reporting Summary

Nature Portfolio wishes to improve the reproducibility of the work that we publish. This form provides structure for consistency and transparency in reporting. For further information on Nature Portfolio policies, see our Editorial Policies and the Editorial Policy Checklist.

Statistics

For all statistical analyses, confirm that the following items are present in the figure legend, table legend, main text, or Methods section.

n/a	Confirmed	
☐	☒	The exact sample size (*n*) for each experimental group/condition, given as a discrete number and unit of measurement
☐	☒	A statement on whether measurements were taken from distinct samples or whether the same sample was measured repeatedly
☐	☒	The statistical test(s) used AND whether they are one- or two-sided<br>*Only common tests should be described solely by name; describe more complex techniques in the Methods section.*
☒	☐	A description of all covariates tested
☒	☐	A description of any assumptions or corrections, such as tests of normality and adjustment for multiple comparisons
☐	☒	A full description of the statistical parameters including central tendency (e.g. means) or other basic estimates (e.g. regression coefficient) AND variation (e.g. standard deviation) or associated estimates of uncertainty (e.g. confidence intervals)
☐	☒	For null hypothesis testing, the test statistic (e.g. *F*, *t*, *r*) with confidence intervals, effect sizes, degrees of freedom and *P* value noted<br>*Give P values as exact values whenever suitable.*
☒	☐	For Bayesian analysis, information on the choice of priors and Markov chain Monte Carlo settings
☒	☐	For hierarchical and complex designs, identification of the appropriate level for tests and full reporting of outcomes
☒	☐	Estimates of effect sizes (e.g. Cohen's *d*, Pearson's *r*), indicating how they were calculated

Our web collection on statistics for biologists contains articles on many of the points above.

Software and code

Policy information about availability of computer code

Data collection	Cyro-EM data was collected in a Titan Krios cryo-electron microscope (Thermo Fisher Scientific) with GIF energy filter (Gatan)
Data analysis	MotionCor2 1.1.0; CTFFIND4; COOT 0.8.9.2; Phenix 1.19.2-4158; UCSF Chimera 1.11.2; PyMOL 1.7.0.1; Graphpad Prism 8.

For manuscripts utilizing custom algorithms or software that are central to the research but not yet described in published literature, software must be made available to editors and reviewers. We strongly encourage code deposition in a community repository (e.g. GitHub). See the Nature Portfolio guidelines for submitting code & software for further information.

Data

Policy information about availability of data

All manuscripts must include a data availability statement. This statement should provide the following information, where applicable:

- Accession codes, unique identifiers, or web links for publicly available datasets
- A description of any restrictions on data availability
- For clinical datasets or third party data, please ensure that the statement adheres to our policy

The cryo-EM structures of the G5C decamer and protomer were deposited into PDB under accession numbers 7XDT (DOI: 10.2210/pdb7XDT/pdb) and 7XGR (DOI: 10.2210/pdb7XGR/pdb), respectively. The cryo-EM density maps were deposited in the Electron Microscopy Data Bank under accession numbers EMD-33152 (www.ebi.ac.uk/pdbe/entry/emdb/EMD-14119) and EMD-33187 (www.ebi.ac.uk/pdbe/entry/emdb/EMD-33187). Source data are provided with this paper. All data supporting the study are available within the paper and its Supplementary Information file.

Field-specific reporting

Please select the one below that is the best fit for your research. If you are not sure, read the appropriate sections before making your selection.

☒ Life sciences ☐ Behavioural & social sciences ☐ Ecological, evolutionary & environmental sciences

For a reference copy of the document with all sections, see nature.com/documents/nr-reporting-summary-flat.pdf

Life sciences study design

All studies must disclose on these points even when the disclosure is negative.

Sample size	No statistical methods were used to predetermine sample sizes. Cryo-EM data sample size was chosen based on the sufficient number of images and particles to obtain a high resolution reconstruction.
Data exclusions	No Data exclusions
Replication	Experiments were repeated independently at least two times. Replicate experiments were sucessful.
Randomization	Sample randomization is not relevant to our study, because for structure determination, the best grid (sample) has to be used for data collection and structure determination. Structure determination was performed following standard procedures.
Blinding	Blinding is not relevant to cryo-EM data collection or structure analysis. Blinding is not applicable for in vitro biochemical or in vivo translation assay.

Reporting for specific materials, systems and methods

We require information from authors about some types of materials, experimental systems and methods used in many studies. Here, indicate whether each material, system or method listed is relevant to your study. If you are not sure if a list item applies to your research, read the appropriate section before selecting a response.

Materials & experimental systems

n/a	Involved in the study	
☐	☒	Antibodies
☐	☒	Eukaryotic cell lines
☒	☐	Palaeontology and archaeology
☒	☐	Animals and other organisms
☒	☐	Human research participants
☒	☐	Clinical data
☒	☐	Dual use research of concern

Methods

n/a	Involved in the study	
☒	☐	ChIP-seq
☒	☐	Flow cytometry
☒	☐	MRI-based neuroimaging

Antibodies

Antibodies used	Anti-Xpress: Invitrogen (ThermoFisher Scientific), catalog R910-25, monoclonal, lot 2190234. The dilution for anti-Xpress antibody is 1:2000.<br>Clone name for anti-Xpress: Invitrogen, reference 46-0528; lot 2190234. Mouse monoclonal IgG1. RRID: AB_2556552.<br>Anti-Tubulin: SIGMA, monoclonal DM1A (ascites fluid), catalog T9026, lot 096k4777. The dilution for anti-tubulin antibody is 1:4000.<br>Secondary antibody: Goat anti-mouse (H+L), Invitrogen (Thermo Fisher Scientific), catalog 32430, lot VJ313743. The dilution for secondary antibody is 1:2000.
Validation	Anti-Xpress was validated in Western blot against 100 ng of an E. coli expressed fusion protein containing the Xpress epitope. The Xpress synthetic peptide sequence is Asp-Leu-Tyr-Asp-Asp-Asp-Asp-Lys.<br>Anti-Tubulin recognizes an epitope located at the C-terminal end of the α-tubulin isoform in a variety of organisms (chicken, kangaroo rat, sea urchin, rat, Chlamydomonas, bovine, human, African green monkey, mouse).

Eukaryotic cell lines

Policy information about cell lines

Cell line source(s)	HEK293 cells (ATCC, CRL-1573)
Authentication	The cell lines were not authenticated
Mycoplasma contamination	The cell lines tested negative for mycoplasms test

March 2021

Commonly misidentified lines
(See ICLAC register)

No commonly misidentified lines were used.
